# The effects of a lipid‐based nutrient supplement and antiretroviral therapy in a randomized controlled trial on iron, copper, and zinc in milk from HIV‐infected Malawian mothers and associations with maternal and infant biomarkers

**DOI:** 10.1111/mcn.12503

**Published:** 2017-08-29

**Authors:** Daniela Hampel, Setareh Shahab‐Ferdows, Erik Gertz, Valerie L. Flax, Linda S. Adair, Margaret E. Bentley, Denise J. Jamieson, Gerald Tegha, Charles S. Chasela, Debbie Kamwendo, Charles M. van der Horst, Lindsay H. Allen

**Affiliations:** ^1^ USDA, ARS Western Human Nutrition Research Center Davis California USA; ^2^ Department of Nutrition University of California Davis California USA; ^3^ Gillings School of Global Public Health University of North Carolina Chapel Hill North Carolina USA; ^4^ Centers for Disease Control and Prevention Atlanta Georgia USA; ^5^ UNC Project Lilongwe Malawi; ^6^ School of Public Health University of Witwatersrand Johannesburg South Africa; ^7^ School of Medicine University of North Carolina Chapel Hill North Carolina USA

**Keywords:** copper, haemoglobin status, HIV, human milk, iron, zinc

## Abstract

We evaluated effects of antiretroviral (ARV) therapy and lipid‐based nutrient supplements (LNSs) on iron, copper, and zinc in milk of exclusively breastfeeding HIV‐infected Malawian mothers and their correlations with maternal and infant biomarkers. Human milk and blood at 2, 6, and 24 weeks post‐partum and blood during pregnancy (≤30 weeks gestation) were collected from 535 mothers/infant‐pairs in the Breastfeeding, Antiretrovirals, and Nutrition study. The participants received ARV, LNS, ARV and LNS, or no intervention from 0 to 28 weeks post‐partum. ARVs negatively affected copper and zinc milk concentrations, but only at 2 weeks, whereas LNS had no effect. Among all treatment groups, approximately 80–90% of copper and zinc and <50% of iron concentrations met the current adequate intake for infants at 2 weeks and only 1–19% at 24 weeks. Pregnancy haemoglobin was negatively correlated with milk iron at 2 and 6 weeks (*r* = −.18, *p* < .02 for both). The associations of the milk minerals with each other were the strongest correlations observed (*r* = .11–.47, *p* < .05 for all); none were found with infant biomarkers. At 2 weeks, moderately anaemic women produced milk higher in iron when ferritin was higher or TfR lower. At 6 weeks, higher maternal α‐1‐acid glycoprotein and C‐reactive protein were associated with higher milk minerals in mildly anaemic women. Infant TfR was lower when milk mineral concentrations were higher at 6 weeks and when mothers were moderately anaemic during pregnancy. ARV affects copper and zinc milk concentrations in early lactation, and maternal haemoglobin during pregnancy and lactation could influence the association between milk minerals and maternal and infant iron status and biomarkers of inflammation.

## 1 INTRODUCTION

Human milk is thought to ensure a balanced and adequate supply of nutrients to infants and is recommended by the World Health Organization (WHO) as the sole food source for the first 6 months of life (Brown, Engle‐Stone, Krebs, & Peerson, 2009; World Health Organization, 2010). Maternal iron, copper, and zinc intake and status are assumed to have no effect on human milk minerals (Domellöf, Lönnerdal, Dewey, Cohen, & Hernell, 2004; Mello‐Neto et al., 2013; Montalbetti, Dalghi, Albrecht, & Hediger, 2014; Moser et al., 1988; Picciano, 2001; Vuori, Mäkinen, Kara, & Kuitunen, 1980); however, Krebs, Hambidge, Jacobs, and Rasbach (1985) reported that milk zinc concentrations are affected by maternal zinc intake within a physiological range and effects of low maternal intakes are most apparent with prolonged lactation. Even though the total concentration of iron, copper, and zinc in human milk is low and decreases throughout lactation, their high bioavailability is assumed to ensure adequate supply during the first 6 months (Dorea, 2000). Nevertheless, iron and zinc deficiencies in the perinatal period remain a public health concern, particularly in low‐income countries, including Malawi (Domellöf et al., 2004; Gibson & Huddle, 1998; National Statistical Office & ICF, 2017), causing symptoms such as diarrhoea, pneumonia, suboptimal growth and development, normocytic hypochromic anaemia, or even osteoporosis (Black et al., 2008; Dauncy, Shaw, & Urman, 1977).

In the context of maternal HIV infection, breastfeeding is recommended for at least 12 months, with continued breastfeeding up to 24 months, whereas maternal antiretroviral (ARV) therapy is provided to reduce the risk of mother‐to‐child HIV transmission (World Health Organization, 2016). However, higher energy and nutrient requirements due to breastfeeding and HIV infection might increase the risk of nutrient deficiencies for mother and infant (de Pee & Semba, 2010; Kayira et al., 2012). Our previous research has shown that ARVs negate the positive effects of maternal lipid‐based nutrient supplement (LNS) consumption during lactation on B‐vitamin concentrations in maternal plasma and human milk (Allen et al., 2015; Flax et al., 2015). Negative ARV effects have also been reported on vitamin D and B12 status (Childs, Welz, Samarawickrama, & Post, 2012; Paltiel, Falutz, Veilleux, Rosenblatt, & Gordon, 1995; Woods et al., 2003). Thus, a potential impact of ARVs on iron, copper, and zinc concentrations in milk is possible and remains to be determined.

We analysed samples from a randomized controlled trial, in which lactating women were assigned to either ARV or LNS, ARV and LNS, or no intervention, to (a) examine the effects of ARV and LNS on iron, copper, and zinc concentrations in milk; (b) compare the concentration of each mineral analysed to the values that were used to set the adequate intake (AI) for infants 0–6 months (Institute of Medicine, 2001); (c) assess correlations among the milk nutrients analysed (iron, copper, zinc, and fat); and (d) examine relationships between milk minerals and maternal and infant characteristics and mineral and inflammation status using maternal and infant biomarkers.

**Key messages**

- Maternal supplementation with lipid‐based nutrient supplements during lactation had no effect on iron, copper, and zinc concentrations in human milk.
- Maternal antiretroviral therapy negatively affected copper and zinc concentrations in human milk in early lactation.
- Milk mineral concentrations quickly decreased during lactation with less than 50% of samples with adequate iron concentrations at 2 weeks.
- The strongest correlations throughout lactation were found among the milk minerals and milk fat.
- Maternal Hb during pregnancy or lactation could influence the relationship between milk minerals and maternal and infant biomarkers for inflammation and iron stores.

## 2 METHODS AND MATERIALS

### 2.1 Participants and sample collection

The participants of the Breastfeeding, Antiretrovirals, and Nutrition (BAN) study included HIV‐infected women recruited 2004–2009 from four antenatal clinics in Lilongwe, Malawi. Details of the recruitment and study design have been previously described (Chasela et al., 2010; Ferguson et al., 2009; Kayira et al., 2012; van der Horst et al., 2009). Informed consent was obtained from all mothers. Based on the WHO guidelines at the time of the study, participants were encouraged to exclusively breastfeed for 24 weeks followed by rapid weaning from 24 to 28 weeks.

At delivery, mothers and their infants were randomized to a two‐arm (LNS, no‐LNS) by three‐arm (maternal ARV, infant ARV, or neither) factorial design, which was condensed to four maternal intervention groups (Figure S1). Infant ARV treatments were not considered as this report focuses on breast milk minerals, and infant treatment would not influence human milk status. Because the LNS purposely included provision of energy and macronutrients, and micronutrients, neither participants nor physicians could be blinded to the study arm. Outcome assessors and sample and data analysts were blinded to the treatment allocation. The screening and enrollment process was conducted by the study nurses; a data manager produced the random distribution. The study pharmacist assigned the participants to the intervention using sequentially numbered envelopes and handed out ARV and LNS during the regular study visits at 0, 1, 2, 4, 6, 8, 12, 18, 21, 24, and 28 weeks post‐partum. The women were instructed to consume two sachets of the LNS per day produced by Nutriset, France, http://www.nutriset.fr (Table S1). The LNS provided 0.3 mg copper, 19 mg zinc, and 15 mg iron equivalent to 23, 158, and 168% of the U.S.–Canada Recommended Dietary Allowances for copper, zinc, and iron, respectively (Institute of Medicine, 2001). The maternal ARV regimen consisted of zidovudine (300 mg) and lamivudine (150 mg; Combivir®, GlaxoSmithKline) plus either nevirapine (until January 2005), nelfinavir (January 2005–February 2006) or lopinavir (200 mg) and ritonavir (50 mg; Kaletra; Abbott; February 2006 until study completion).

Reports of maternal LNS consumption were obtained at 1, 4, 8, 12, and 21 weeks. Maternal haemoglobin (Hb) was assessed at ≤30 weeks gestation during the prenatal screening, and maternal and infant venous blood was collected at birth, 2, 6, 12, 18, and 24 weeks for further Hb measurements. Plasma for additional biomarker analysis was separated from red blood cells within 60 min and stored at −70 °C in 1 ml aliquots. Ten millilitres of human milk was collected during the regular study visits at 2 and 24 weeks (*n* = 366), or 6 and 24 weeks (*n* = 169) when a 2‐week milk or infant plasma sample was insufficient for micronutrient analysis. The milk was manually expressed by the study participant yielding an opportunistic sample that was immediately frozen after collection. The number of participants with available milk, maternal, and infant plasma samples predetermined the sample size (*n* = 535). Participants were preferentially selected if they provided anthropometry and dietary intake data. If the infants became HIV‐positive or were multiple births, they were excluded from the subset. Details of the primary study's sample size calculations have been previously described (van der Horst et al., 2009).

Samples were initially shipped on dry ice to the Centers for Disease Control and Prevention in Atlanta and stored at −80 °C until distribution to the USDA, ARS Western Human Nutrition Research Center in Davis, California, for analysis.

### 2.2 Biochemical analyses

Iron, copper, and zinc were analysed in 500 μl of whole human milk. One hundred fifty microlitres of the 1.00 ppm calibration standard in nitric acid (HNO_3_, 3.2N, for trace analysis), 2.25 ml HNO_3_ (3.2N), and 100 μl peroxide (for trace analysis) were added to a final volume of 3.0 ml. Samples were mixed and microwaved for 15 min at 90 °C (600 W, 5 min ramp from ambient temperature to 90 °C, temperature controlled). After a 5‐min cooling step, the samples were centrifuged for 10 min at 2,750 × g (4 °C) after which they were transferred into the 8 ml polypropylene tubes (13 × 100 mm, Perfector Scientific, Fair Lawn, NJ, USA) for analysis or capped and stored at 4 °C until analysis. The laboratory internal control BLS (National Institute of Standards and Technology standard reference material (SRM)1577c bovine liver, NIST, Gaithersburg, MD) and the 0.2 ppm standard, analysed with each sample set, revealed an inter‐assay variation of 1.5–7.0% and an intra‐assay variation of 0.1–10.8% over a period of 6 weeks (*n* = 25). The calibration curve for each mineral consisted of a blank and 10 standard solutions in a linear range of 0.0125–2.000 ppm prepared in 3.2N HNO_3_. Due to the difference in concentration of each analyte in human milk, the actual standard concentrations used for each mineral were as follows: Cu: 0.0125–1.000 ppm, Fe: 0.025–1.000 ppm, and Zn: 0.0125–2.000 ppm. Mean recovery rates for copper, iron, and zinc were 92.6 ± 5.7%, 89.4 ± 7.2%, and 94.7 ± 6.8%, respectively. The analyses were conducted using a Varian Vista AX CCD Simultaneous ICP‐AES (Folsom, CA, USA) operated by ICPExpert software. Samples were digested using the CEM microwave accelerated reaction system (MARS 5; Matthews, NC, USA). The milk fat was measured using the Creamatocrit Plus (Medela, McHenry, IL, USA) according to the manufacturer's protocol. The tube reader's accuracy was validated using the Medela control strip.

The inflammatory markers α‐1‐acid glycoprotein (AGP) and C‐reactive protein (CRP) and soluble transferrin receptors (TfR) in maternal and infant plasma were analysed by a COBAS® INTEGRA 400 plus multianalyte analyser (Roche Diagnostics, Mannheim, Germany). Ferritin concentrations were determined using IRMA Ferritin Coat‐a‐Count radioimmunoassay (Siemens Health Care Diagnostics, Tarrytown, NY, USA), and Hb was measured using the AcT or AcT 5‐part Differential Analyser (Beckman Coulter, Fullerton, CA, USA).

### 2.3 Ethics

This research was approved by the Malawian National Health Science Research Committee, the Institutional Review Boards at the University of North Carolina at Chapel Hill, the U.S. Centers for Disease Control and Prevention, and the University of California, Davis (Clinical http://Trials.gov; NCT00164762). The trial was monitored for safety and efficacy by the National Institute of Allergy and Infectious Diseases Vaccine and Prevention Data and Safety Monitoring Board.

### 2.4 Statistical methods

Because milk sample collection started 2 to 6 weeks after the LNS intervention, no true baseline concentrations were available for statistical analyses (Allen et al., 2015). A separate binary variable, hereafter referred to FTP (first time point), was used to control for whether the FTP of sample collection occurred at 2 (FTP2) or 6 weeks (FTP6). The outcomes were concentrations of iron, copper, and zinc in human milk, within the four defined groups (ARV, LNS, ARV/LNS, and control). SAS® for Windows Release 9.4 (SAS Institute, Cary, NC, USA) was used for all statistical analyses. Square root transformations were performed on all outcome variables to normalize the distributions. The original hypothesis focused on the effect of LNS supplementation on outcomes. However, preliminary data revealed significant effects due to ARV for some of the minerals, so the hypothesis was modified to include possible main effects and modifying effects of ARV. Mixed models repeated measures analysis of variance was used to fit a four‐factor model, which included LNS, ARV, and FTP as between‐subject main effects, time as a within‐subject main effect, all two‐, three‐, and four‐factor interactions, and a random effect of subject assuming an unstructured covariance matrix. When there was no significant interaction between treatment and FTP, treatment was assessed as a main effect only.

All analyses described in this paragraph were conducted using pooled milk values for each mineral from all treatment groups. Iron, copper, and zinc milk concentrations were significantly lower at 6 weeks than at 2 weeks; thus, to estimate the correlations of the breast milk iron, copper, and zinc concentrations with maternal and infant biomarkers and characteristics, we analysed the data separately by FTP (2 or 6 weeks) subgroup. Pearson correlations procedure was used. Concentrations of maternal ferritin, maternal CRP, maternal TfR, infant ferritin, infant CRP, infant AGP, and maternal age were log‐transformed; infant TfR and birthweight were square‐root transformed, and maternal AGP, body mass index (BMI), and infant Hb were inverted (1/x) to achieve normal distributions. Inverted variables were multiplied by −1 to preserve direction. Pregnancy Hb, maternal Hb, and infant birth length were normally distributed and not transformed. Spearman correlations were used to explore relationships of breast milk iron, copper, and zinc concentrations with non‐normalized maternal and infant characteristics including marital status, literacy, education, parity, famine season (August to March), and infant sex. Linear regression analysis was used to examine associations (a) between maternal iron status (ferritin, TfR) and inflammation (AGP, CRP) biomarkers on human milk iron, copper, and zinc at 2, 6, and 24 weeks, stratified by anaemia status during pregnancy (non‐anaemic: Hb ≥ 110 g/L, mildly anaemic: Hb 100–109 g/L, moderately anaemic: Hb 70–99 g/L, and severely anaemic: Hb ≤ 70 g/L) and lactation (non‐anaemic: Hb ≥ 120 g/L, mildly anaemic: Hb 110‐119 g/L, moderately anaemic: Hb 80‐109 g/L, and severely anaemic: Hb ≤ 80 g/L; World Health Organization, 2011) and (b) between maternal iron status biomarkers (ferritin, TfR, Hb, and pregnancy Hb) on human milk iron, copper, and zinc at 2, 6, and 24 weeks, stratified by inflammation status (AGP > 1 g/L and CRP > 5 mg/L).

For all procedures, *p* values < .05 were considered to be statistically significant. Significant differences in the distribution of categorical variables were examined using the chi‐square test. Significant changes in milk adequacy (based on AI) over time (2, 6, and 24 weeks) were tested using the GLIMMIX procedure.

The measured human milk iron, copper, and zinc concentrations at each time point (TP), FTP of milk collection, and treatment group were also compared to milk values from well‐nourished women used by the Institute of Medicine to set AIs for infants from 0–6 months of age (Institute of Medicine, 2001).

## 3 RESULTS

### 3.1 Maternal characteristics at FTP

The characteristics of the participants were similar among treatment groups at the FTP of human milk collection (Table 1). Significant differences were only observed for the CD4 counts with higher counts for the groups that received ARV. The median BMI was within the normal range and the overall compliance with supplementation and ARV treatment was high. In the adherence reports collected over five follow‐up visits, the mothers self‐reported that they took their prescribed ARV treatment 89% of the time and LNS supplement 92% of the time. The self‐reported frequency of exclusive breastfeeding was 96% at 21 weeks post‐partum (Chasela et al., 2010; Kayira et al., 2012).

**Table 1:** Characteristics of mothers in the BAN study at the first time point of collection (2/6 weeks)

	Treatment group								
	Control		LNS		ARV		ARV + LNS		
Characteristics	*n*	Median (IQR)	*n*	Median (IQR)	*n*	Median (IQR)	*n*	Median (IQR)	*p* value^f^
Age (y)	176	25.5 (22.9–29.8)	183	26.2 (22.2–29.9)	85	27.0 (24.0–29.4)	90	25.0 (22.9–30.0)	.58
Literacy (%)	172	78.5	179	75.4	81	76.5	85	77.6	.92
Post‐primary education (%)	177	36.7	185	37.8	85	35.3	90	32.2	.75
Married (%)	177	90.4	185	90.3	85	89.4	90	92.2	.39
Primiparous (%)	177	18.1	185	17.8	85	11.8	90	16.7	.59
Vaginal delivery (%)	177	96.6	185	95.7	85	96.5	90	94.4	.91
Anthropometric measurements									
Height (cm)	177	157 (154–160)	185	155 (152–159)	85	157 (154–160)	90	157 (154–161)	.05
Weight (kg)	177	55.5 (50.0–60.3)	185	54.4 (50.3–59.1)	85	54.6 (50.5–60.2)	90	54.5 (50.2–60.4)	.84
BMI (kg/m^2^)	177	22.4 (20.7–24.1)	185	22.3 (20.9–24.2)	85	22.3 (20.3–24.3)	90	22.2 (20.8–23.7)	.55
BMI < 18.5 (%)^a^	177	0.0	185	3.2	85	4.7	90	5.6	.031
BMI 18.5–24.9 (%)	177	83.1	185	77.3	85	75.3	90	78.9	.43
BMI 25–29.9 (%)	177	15.3	185	16.2	85	15.3	90	15.6	.99
BMI ≥ 30 (%)	177	1.7	185	3.2	85	4.7	90	0.0	.17
Laboratory measurements									
AGP (g/L)	177	1.2 (0.9–1.5)	185	1.2 (0.9–1.4)	85	1.2 (1.0–1.6)	90	1.2 (0.9–1.4)	.65
AGP > 1 g/L (%)^b^	177	27.1	122	35.7	85	40.0	90	35.6	.14
CRP (mg/L)	177	3.3 (1.3–9.1)	185	3.2 (1.5–7.5)	85	2.8 (1.5–10.0)	90	2.7 (1.4–6.1)	.43
CRP > 5 mg/L (%)^c^	177	3.4	185	3.8	85	1.2	90	3.3	.71
AGP > 1gL and CRP > 5 mg/L (%)	177	39.0	185	30.3	85	35.3	90	25.6	.12
Ferritin	177	30.2 (15.8–56.0)	185	25.5 (13.0–49.7)	85	26.0 (16.0–55.1)	90	26.2 (12.8–68.5)	.85
TfR	177	4.9 (3.7–6.4)	185	5.2 (3.9–7.2)	85	5.1 (3.8–6.6)	90	5.2 (3.7–7.0)	.43
Haemoglobin (g/L)	177	122 (111–131)	185	121 (111–131)	85	121 (107–126)	90	120 (111–131)	.28
Iron deficiency (%)^c^	177	5.1	185	7.6	85	10.6	90	13.3	.10
Iron deficiency anaemia (%)^d^	177	3.4	185	5.9	85	7.1	90	13.3	.020
Anaemic during pregnancy (%)^e^	177	53.7	185	56.2	85	51.8	90	47.8	.58
CD4 count (cells/μl)	159	465 (319–665)	170	500 (337–738)	78	616 (439–780)	85	607 (412–796)	<.001

*Note*. Characteristics are provided for the subsample (*n* = 535). Anaemic during pregnancy: haemoglobin < 110 g/L; ARV, antiretroviral; AGP = α‐1‐acid glycoprotein; BAN, Breastfeeding, Antiretrovirals, and Nutrition; CRP = C‐reactive protein; IQR = interquartile range; LNS, lipid‐based nutrient supplement; TfR, soluble transferrin receptors.

a BMI categories: <18.5, underweight; 18.5–24.9, normal; 25–29.9, overweight; >30, obese.

b Elevated AGP as an indicator for chronic inflammation.

Elevated CRP as an indicator acute inflammation.

c Iron deficiency defined as ferritin <15 μg/L and TfR > 8.5 mg/L.

d Iron deficiency anaemia defined as Hb < 120 g/L, ferritin <15 μg/L, and TfR > 8.5 mg/L.

e Defined as Hb < 110 g/L.

f *p* value: chi‐square test for categorical variables and generalized linear models for continuous variables.

### 3.2 Treatment effects and time interactions

The mixed model analysis revealed that the concentrations of the three minerals changed over time (TP) and differed by FTP (*p* < .02 for all). For iron, no interactions by treatment (ARV and LNS) or with treatment and TP and/or FTP were significant indicating that the effects of ARV and LNS at 2, 6, and 24 weeks were not different from each other (Table S2). Therefore, pooled data for all treatment groups and all TPs combined were used for further statistical analyses involving iron. ARV by TP by FTP interactions were significant for copper (*p* = .034), whereas ARV by FTP interactions were significant for zinc (*p* = .031). Thus, to evaluate the main effects, samples from 2, 6, and 24 weeks were analysed separately for copper and zinc.

The reduction in milk iron concentrations between 2, 6, and 24 weeks was independent of treatment. ARV by LNS interactions were not significant for any of the minerals analysed nor were LNS and ARV main effects significant for iron (Table 2). The mixed model analysis for copper and zinc at 2, 6, and 24 weeks showed that ARV treatment significantly and negatively affected milk copper and zinc concentrations (*p* = .044 and *p* = .014) but only at the initial 2 weeks TP.

**Table 2:** Median concentrations and interquartile ranges (IQR) of iron, copper, and zinc and main effects of ARV and LNS of iron, copper, and zinc at 2, 6, and 24 weeks in breast milk of HIV‐infected Malawian mothers assigned to one of the four treatment arms in the BAN study

Treatment		Iron			Copper			Zinc		
	Week	2	6	24	2	6	24	2	6	24
Control	*n*	118	59	177	118	59	177	118	59	177
mg/L	Median	0.342	0.250	0.190	0.387	0.223	0.143	3.57	2.49	1.16
	IQR	0.23–0.50	0.17–0.40	0.13–0.30	0.32–0.47	0.20–0.31	0.10–0.18	3.01–4.21	2.07–3.06	0.75–1.57
LNS	*n*	140	45	185	140	45	185	140	45	185
mg/L	Median	0.331	0.230	0.188	0.372	0.247	0.127	3.59	2.39	1.05
	IQR	0.25–0.49	0.14–0.37	0.11–0.27	0.32–0.45	0.21–0.30	0.09–0.17	2.92–4.31	1.89–3.10	0.70–1.56
ARV	*n*	53	31	85	53	31	85	53	31	85
mg/L	Median	0.337	0.297	0.157	0.379	0.273	0.151	3.16	2.46	1.05
	IQR	0.24–0.46	0.16–0.45	0.11–0.27	0.32–0.45	0.23–0.35	0.12–0.207	2.75–3.63	1.95–3.20	0.75–1.54
ARV/LNS	*n*	55	34	90	55	34	90	55	34	90
mg/L	Median	0.301	0.265	0.210	0.341	0.258	0.141	3.40	2.47	1.13
	IQR	0.21–0.43	0.13–0.36	0.13–0.27	0.29–0.40	0.21–0.33	0.10–0.18	2.88–3.85	1.95–2.94	0.72–1.57
Treatment effects^a^										
LNS	LS mean	0.516			0.615	0.504	0.362	1.87	1.53	1.04
No LNS	LS mean	0.514			0.621	0.508	0.376	1.86	1.57	1.07
*p* value		.86			.49	.80	.08	.74	.34	.14
ARV	LS mean	0.505			0.603	0.519	0.378	1.82	1.55	1.06
No ARV	LS mean	0.520			0.624	0.498	0.364	1.89	1.55	1.06
*p* value		.23			.044	.16	.11	.014	.95	.95

*Note*. ARV = antiretrovirals; BAN, Breastfeeding, Antiretrovirals, and Nutrition; LNS, lipid‐based nutrient supplement; LS Mean = least‐square mean.

a Treatment effects (LNS–no LNS; ARV–no ARV) were tested by mixed model repeated measures analysis of variance. Treatment effects for iron are derived from a pooled sample (2, 6, and 24 weeks combined).

### 3.3 Comparison of milk iron, copper, and zinc concentrations with values used to set the AI for infants 0–6 months

At 2 weeks, 38–48% of all milk samples met the milk iron concentrations assumed from reported data used to estimate the AI for infants from 0 to 6 months (Table 3). Depending on treatment group, approximately 89–94% and 78–88% of the samples had copper and zinc concentrations at 2 weeks that corresponded to AI values (Institute of Medicine, 2001).

**Table 3:** Percentage [%] of milk samples from the subsample of the BAN study that meet the values used to set the adequate intake (AI) for each mineral, by collection time and treatment group

Mineral	AI	Control		LNS		ARV		ARV/LNS		
[mg/L]	[mg/L]	[%]	(*n*)	[%]	(*n*)	[%]	(*n*)	[%]	(*n*)	*p* value^b^
Iron										
2 weeks	0.346	48	(57/118)	44	(61/140)	45	(24/53)	38	(21/55)	0.62
6 weeks		32	(19/59)	27	(12/45)	35	(11/31)	32	(11/34)	0.83
24 weeks		18	(31/177)	16	(30/185)	11	(9/85)	19	(15/80)	0.54
*p* value^a^		< 0.024								
Copper										
2 weeks	0.256	91	(107/118)	94	(132/140)	92	(49/53)	89	(49/55)	0.64
6 weeks		39	(23/59)	44	(20/45)	61	(19/31)	50	(17/34)	0.17
24 weeks		9	(15/177)	6	(11/185)	9	(8/85)	5	(4/80)	0.48
*p* value		< 0.001								
Zinc										
2 weeks	2.564	88	(104/118)	88	(123/140)	81	(43/53)	78	(43/55)	0.21
6 weeks		44	(26/59)	40	(18/45)	45	(14/31)	47	(16/31)	0.92
24 weeks		3	(6/177)	1	(2/185)	4	(1/85)	1	(1/85)	0.34
*p* value		< .001								

*Note*. ARV = antiretrovirals; BAN = Breastfeeding, Antiretrovirals, and Nutrition; LNS = lipid‐based nutrient supplement; number of samples given in parentheses (numerator: *n* of samples that met AI, denominator: total *n*).

a *p* values for comparison of proportions of samples with adequate milk iron, copper, zinc concentrations over time (2, 6, 24 weeks; proc glimmix).

b *p* value for comparison of proportions of samples with adequate milk iron, copper, and zinc concentrations based on treatment (LNS, ARV, ARV/LNS, and control; chi‐square test).

Lower breast milk iron, copper, and zinc concentrations were observed at 6 weeks: 27–35% of the milk samples met the AI for iron concentrations and 39–61% and 40–47% the AI for copper and zinc concentrations.

**Figure 1:**
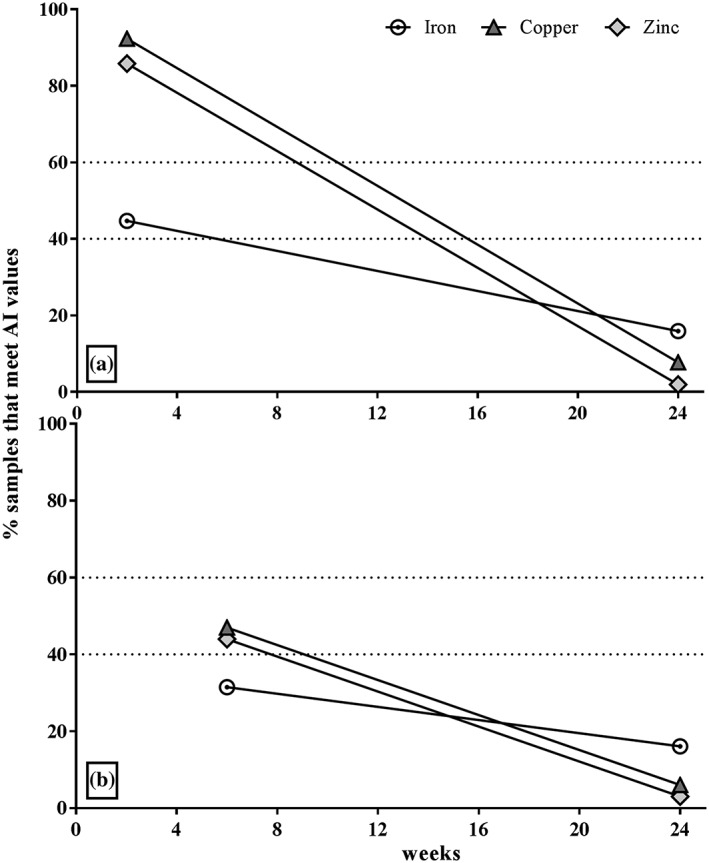
Relative median decrease (%) in human milk iron, copper, and zinc over time compared to AI (values pooled from all treatment groups at each time point of sample collection: (a) FTP2 subgroup (2–24 weeks; *n* = 366); (b) FTP6 subgroup (6–24 weeks; *n* = 169). AI = adequate intake; FTP = first time point

At 24 weeks, only about 11–19% of all samples met the AI estimates for iron. Copper and zinc concentrations were even lower; only 5–9% (copper) and 1–4% (zinc) of the samples met the AI concentrations. Plotting the relative amount of samples that met the AI longitudinally by FTP subgroup (2–24 and 6–24 weeks), copper and zinc followed the same pattern of decrease, whereas the iron AI was met by considerably fewer samples compared to the copper and zinc already at 2 weeks, but then followed a slower rate of decrease (Figure 1).

### 3.4 Correlations of maternal and infant biomarkers and characteristics with breast milk minerals

**Figure 2:**
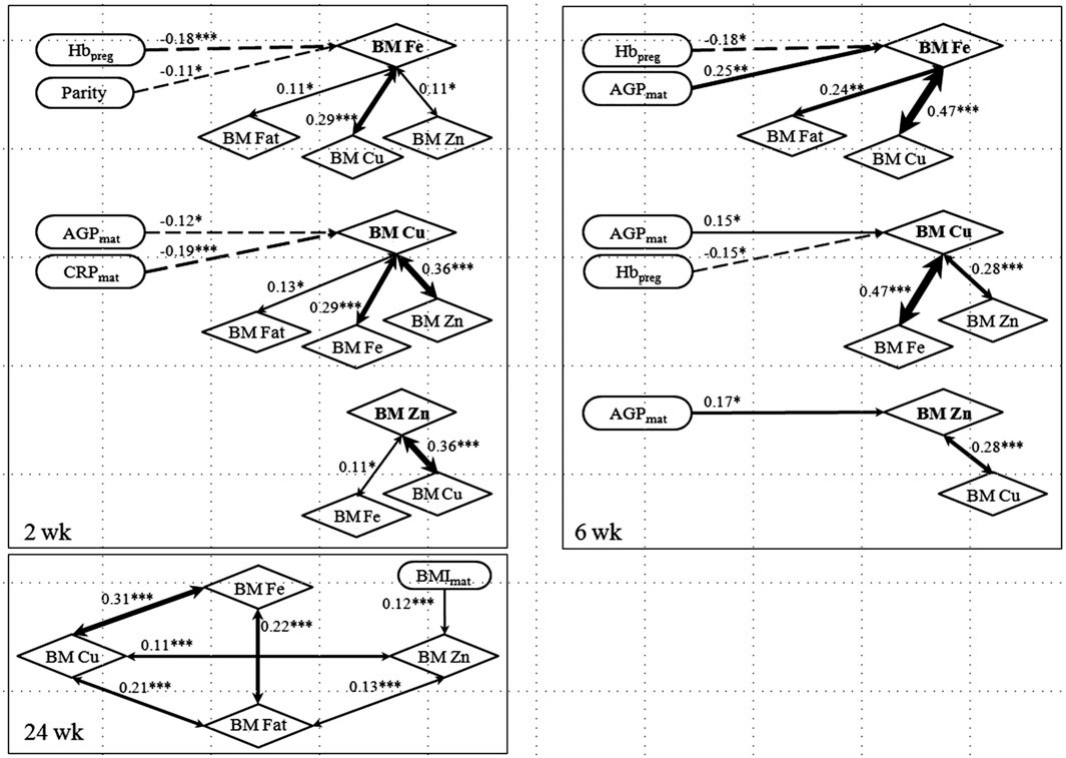
Significant correlations between milk minerals and maternal biomarkers at 2, 6, and 24 weeks. Pearson's correlation coefficients (Spearman correlations coefficient for the characteristics) are shown and level of significance as: *p < .05; **p < .01; ***p < .001. Preg = pregnancy (during screening); Hb = haemoglobin; BM = breast milk; Fe = iron; Cu = copper; Zn = zinc; Mat = maternal; AGP = α‐1‐acid glycoprotein; CRP = C‐reactive protein; BMI = body mass index

Iron, 2 and 6 weeks: At 2 weeks, human milk iron concentrations were significantly correlated with pregnancy Hb (*r* = −.18, *p* < .001) and parity (*r* = −.11, *p* = .031) as well as with breast milk copper (*r* = .29, *p* < .001), zinc (*r* = .11, *p* = .028), and fat (*r* = .11, *p* = .044; Figure 2). At 6 weeks, breast milk iron was correlated with pregnancy Hb (*r* = −.18, *p* = .019), maternal AGP (*r* = −.25, *p* = .001), and breast milk copper (*r* = .47, *p* < .001) and fat (*r* = .24, *p* = .002).

Copper, 2 and 6 weeks: At 2 weeks, human milk copper concentrations were correlated with maternal AGP (*r* = .12, *p* = .021) and CRP (*r* = −.19, *p* < .001) and milk iron, zinc (*r* = .36, *p* < .001), and fat (*r* = .13, *p* = .01; Figure 2). At 6 weeks, milk copper was also correlated with pregnancy Hb (*r* = −.15, *p* = .047), maternal AGP (*r* = −.15, *p* = .049), and breast milk iron and zinc (*r* = .28, *p* < .001).

Zinc, 2 and 6 weeks: At 2 weeks, milk zinc concentrations were significantly correlated only with milk iron and copper. At 6 weeks, human milk zinc was correlated with maternal AGP (*r* = −.17, *p* = .03) and breast milk copper.

Iron, copper, and zinc, 24 weeks: At 24 weeks, neither iron nor copper concentrations were correlated with any of the maternal or infant biomarkers (Figure 2). Only zinc showed a correlation with maternal BMI (*r* = .12, *p* = .006). Significant correlations remained between the analysed human milk minerals and fat: Iron was correlated with copper (*r* = .31, *p* < .001) and fat (*r* = .22, *p* < .001) and copper with zinc (*r* = .11, *p* < .001) and fat (*r* = .21, *p* < .001); additionally, zinc was correlated with fat (*r* = .13, *p* = .003).

None of the infant biomarkers or characteristics were significantly associated with human milk iron, copper, or zinc concentrations at any TP.

### 3.5 Associations of human milk iron, copper, and zinc with maternal and infant biomarkers, stratified by maternal Hb (anaemia status) during pregnancy and lactation

#### 3.5.1 Maternal biomarkers

**Figure 3:**
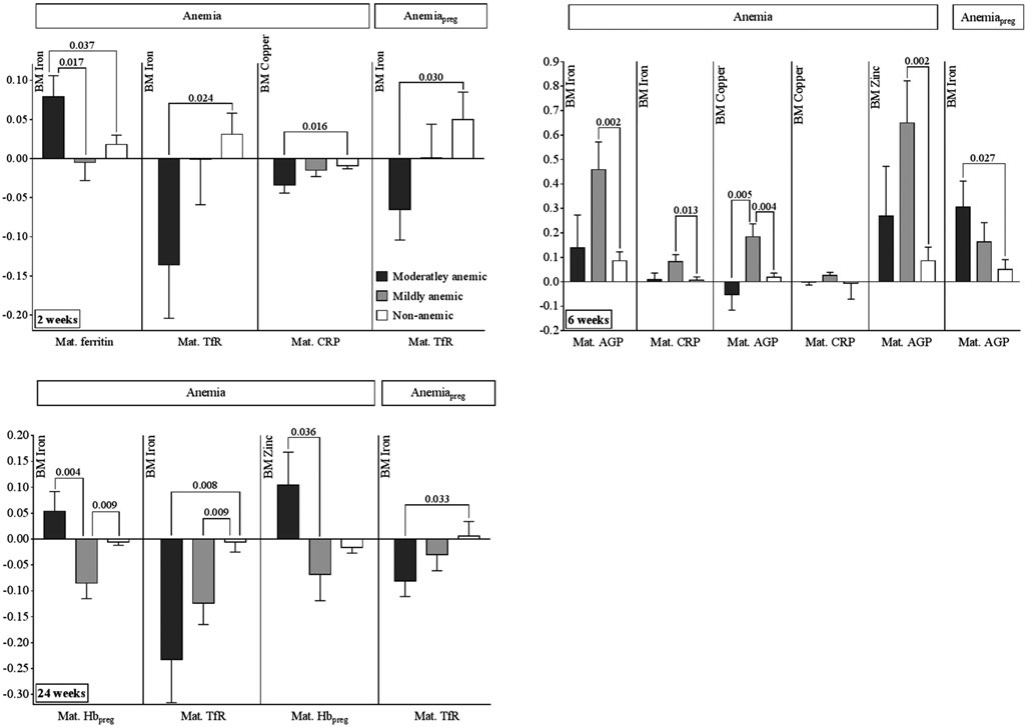
Associations of human milk iron, copper, and zinc with maternal biomarkers stratified by maternal haemoglobin status during pregnancy and lactation (coefficients and *SE*s obtained from regression procedure displayed as bar graphs with error bars). *p* values for comparisons of moderately or mildly anaemic to non‐anaemic group, and comparison of moderately to mildly anaemic group, using linear regression analysis. Anaemia, state of anaemia—moderately, mildly, or non‐anaemic—based on maternal haemoglobin status at indicated week post‐partum (World Health Organization, 2003). Anaemia_Preg_, state of anaemia during pregnancy (moderately, mildly, or non‐anaemic based on maternal Hb during pregnancy, World Health Organization, 2003). BM = breast milk; CRP = C‐reactive protein; AGP = α‐1‐acid glycoprotein; TfR = soluble transferrin receptors; *n* = number of samples (moderately anaemic/mildly anaemic/non‐anaemic): 2 weeks anaemia: 41/49/276; 2 weeks anaemia_Preg_: 91/106/169; 6 weeks anaemia: 13/25/131; 6 weeks anaemia_Preg_: 35/53/81; 24 weeks anaemia: 16/45/476; 24 weeks anaemia_Preg_: 126/160/251; separate models were used for each time point and first time point and anaemia status

2 weeks: When women were moderately anaemic post‐partum, higher ferritin during lactation was associated with higher human milk iron concentrations when compared to mildly anaemic (*p* = .017) and non‐anaemic (*p* = .037, Figure 3) pregnant women, whereas higher TfR in lactation was associated with lower milk iron compared to the non‐anaemic group (*p* = .024). Additionally, higher maternal CRP during lactation was associated with lower milk copper compared to that of non‐anaemic women (*p* = .016). The degree of anaemia in pregnancy did not affect the relationship of the maternal biomarkers to milk zinc concentrations (data not shown). When women were moderately anaemic during pregnancy, higher TfR concentrations post‐partum were associated with lower milk iron concentrations compared to those of non‐anaemic women (*p* = .030).

6 weeks: In women who were mildly anaemic during lactation, higher CRP post‐partum was associated with higher milk iron (*p* = .013) and copper (*p* = .036) compared to non‐anaemic women, whereas higher AGP was associated with higher milk iron (*p* = .002) and zinc (*p* = .002) concentrations. Higher AGP was associated with lower copper concentrations when comparing moderately anaemic post‐partum women to mildly anaemic women (*p* = .004), yet higher AGP was associated with higher copper when comparing mildly anaemic to non‐anaemic post‐partum women (*p* = .005). When women were moderately anaemic during pregnancy, higher post‐partum AGP concentrations at 6 weeks were associated with higher milk iron concentrations compared to concentrations in non‐anaemic women (*p* = .027).

24 weeks: Higher maternal TfR concentrations at 24 weeks were associated with lower milk iron values in moderately (*p* = .008) and mildly (*p* = .009) anaemic women compared to non‐anaemic participants at 24 weeks.

#### 3.5.2 Infant biomarkers

**Figure 4:**
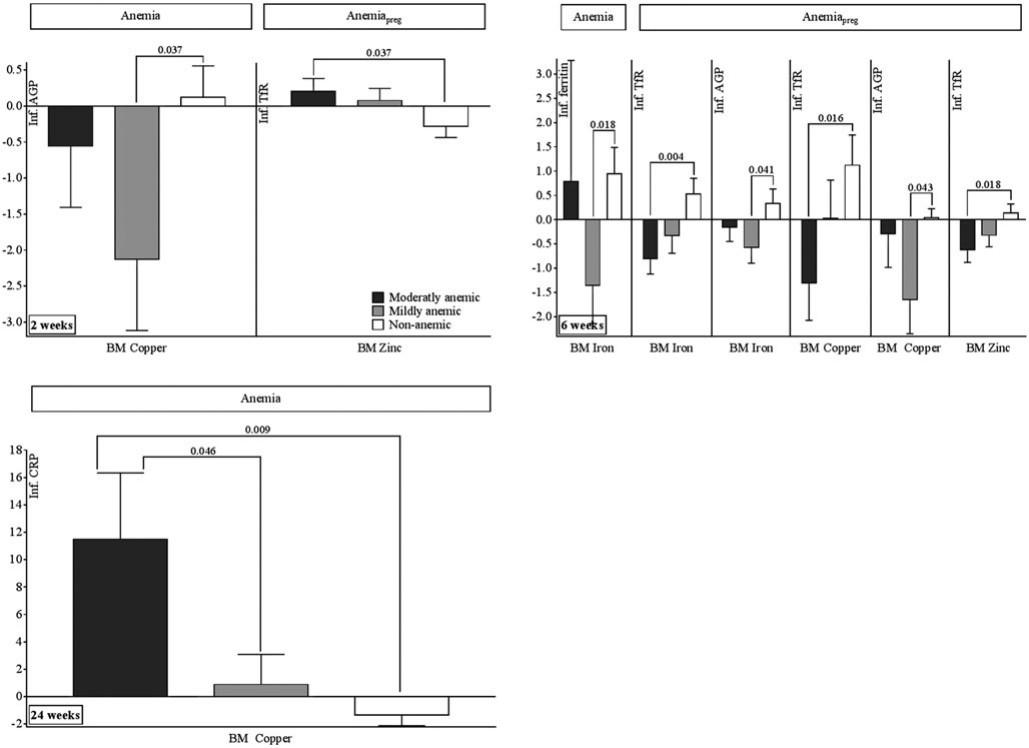
Associations of human milk iron, copper, and zinc with infant biomarkers stratified by maternal haemoglobin status during pregnancy and lactation (coefficients and *SE*s obtained from regression procedure displayed as bar graphs with error bars). *p* values for comparisons of moderately or mildly anaemic to non‐anaemic group, and comparison of moderately to mildly anaemic group, using linear regression analysis. Anaemia, state of anaemia—moderately, mildly, or non‐anaemic—based on maternal haemoglobin status at indicated week post‐partum (World Health Organization, 2003). Anaemia_Preg_, state of anaemia during pregnancy (moderately, mildly, or non‐anaemic based on maternal Hb during pregnancy, World Health Organization, 2003). BM = breast milk; CRP = C‐reactive protein; AGP = α‐1‐acid glycoprotein; TfR = soluble transferrin receptors; *n* = number of samples (moderately anaemic/mildly anaemic/non‐anaemic): 2 weeks anaemia: 41/49/276; 2 weeks anaemia_Preg_: 91/106/169; 6 weeks anaemia: 13/25/131; 6 weeks anaemia_Preg_: 35/53/81; 24 weeks anaemia: 16/45/476; 24 weeks anaemia_Preg_: 126/160/251; separate models were used for each time point and FTP and anaemia status

2 weeks: Higher milk copper was associated with lower AGP at 2 weeks among infants of mothers who were mildly anaemic during lactation, compared to infants of mothers who were not anaemic, (*p* = .037; Figure 4). For those infants whose mothers were moderately anaemic during pregnancy, higher milk zinc was associated with higher infant TfR concentrations compared to infants born to women who were not anaemic during pregnancy (*p* = .037).

6 weeks: When the women had been mildly anaemic during lactation, higher milk iron concentrations were associated with lower infant ferritin (*p* = .018) compared to infants born to non‐anaemic women. When mothers were moderately anaemic during pregnancy, higher milk iron, copper, and zinc concentrations were associated with lower infant TfR compared to women whom were not anaemic (*p*_iron_ = .004, *p*_copper_ = .016, *p*_zinc_ = .018). When women were mildly anaemic during pregnancy, higher milk iron and copper were associated with lower infant AGP at 6 weeks compared to non‐anaemic women during pregnancy (*p*_iron_ = .041, *p*_copper_ = .043).

24 weeks: When mothers were moderately anaemic, increasing copper in milk was associated with increasing infant CRP when compared to mildly (*p* = .046) and non‐anaemic (*p* = .009) women.

### 3.6 Human milk iron, copper, and zinc concentrations and inflammation

In the pooled samples set (*n* = 1,074), iron, copper, and zinc concentrations in the milk of HIV‐infected Malawian mothers were significantly higher (*p* < .001 for all; Table 4) in women with evidence of inflammation (AGP > 1 g/L, CRP > 5 mg/L). Moreover, women with inflammation revealed higher milk iron concentrations at 2 weeks when their pregnancy Hb status was lower, *p* = .015; no inflammation: coeff. (std error): −0.012 (0.010), inflammation: −0.050 (0.013), and showed higher milk zinc concentrations at 6 weeks when their pregnancy Hb status was higher, *p* = .020; no inflammation: coeff. (std error): −0.032 (0.022), inflammation: 0.091 (0.047).

**Table 4:** Median concentrations and interquartile ranges (IQR) of the pooled samples (all TP and FTP) of iron, copper, and zinc in breast milk of HIV‐infected Malawian mothers in the BAN study based on inflammation status

Mineral	No inflammation	Inflammation
*n*	835	239
Iron		
Median, mg/L	0.224	0.325
IQR, mg/L	0.140–0.342	0.212–0.456
*p* value^a^	<.001	
Copper		
Median, mg/L	0.196	0.291
IQR, mg/L	0.129–0.319	0.192–0.375
*p* value	<.001	
Zinc		
Median, mg/L	1.75	2.90
IQR, mg/L	0.988–2.93	1.69–3.74
*p* value	<.001	

*Note*. Inflammation defined as AGP > 1 g/L and CRP > 5 mg/L.

a *p* values obtained from general linear models.

## 4 DISCUSSION

The recommendation to exclusively breastfeed for the first 6 months of life demands an understanding of how maternal micronutrient supplementation and drugs, such as ARVs, affect human milk nutrient concentrations. LNS are commonly used to treat severe acute malnutrition in children; their effectiveness in improving birth and health outcomes in infants and children with moderate malnutrition and their effectiveness in prevention of stunting has also been under evaluation (Adu‐Afarwuah et al., 2015; Prado et al., 2016; Phuka et al. 2009; Thakwalakwa et al., 2010). In the BAN study, maternal LNS mitigated maternal weight loss even when ARVs were given (Kayira et al., 2012) and increased human milk B‐vitamin concentrations, whereas ARVs negatively affected some B‐vitamins. When ARV and LNS were given, ARV diminished the positive LNS effect (Allen et al., 2015). However, beneficial effects of maternal supplementation or fortification on human milk B‐vitamins have been also observed in other studies (Duggan et al., 2014; Shahab‐Ferdows et al., 2015; Siddiqua et al., 2016; Whitfield et al., 2016).

### 4.1 Treatment effects

Human milk iron, copper, and zinc concentrations are thought to be unaffected by maternal supplementation (Domellöf et al., 2004; Montalbetti et al., 2014; Moser et al., 1988; Picciano, 2001; Vuori et al., 1980), which is consistent with our results. Maternal ARV treatment, however, negatively affected milk copper and zinc concentrations at 2 weeks, resulting in a 5–8% reduction of the median copper and zinc concentration available to the infant. These reduced amounts might not be significant to a healthy infant but could impact infants exposed to nutritional deficiencies as they do not possess extensive zinc or copper stores. Moreover, maternal ARV treatment in this study was reflected in the milk, and lamivudine was found in infant plasma (Corbett et al., 2014; Shapiro et al., 2005), observations particularly important in light of the 2016 WHO guidelines that recommend ARV provision to HIV‐infected mothers of HIV‐exposed infants. Certain ARVs provided to the mother are transmissible through human milk, which could impact the infants' ability to fight a possible HIV infection (Zeh et al., 2011). Thus, maternal ARVs are not only transferring to the infant through human milk but also, in our study, affect the transfer of zinc and copper during early lactation, and possibly other nutrients, into milk and subsequently to the infant.

The milk mineral concentrations from these HIV‐infected mothers were comparable to other reported values from healthy women in the United States, Iran, and Brazil (Mahdavi, Nikniaz, & Gayemmagami, 2010; Mello‐Neto et al., 2013; Picciano & Guthrie, 1976). Thus, maternal HIV status might not or only marginally affect milk mineral concentrations. However, another study conducted in the United States revealed considerably higher mineral concentrations in milk from apparently healthy mothers (Feeley, Eitenmiller, Jones, & Barnhart, 1983), whereas women in Vietnam produced milk with higher iron and copper but lower zinc concentrations at 6 months post‐partum (Nakamori et al., 2009).

### 4.2 Comparison of milk iron, copper, and zinc concentrations with values used to set the AI for infants 0–6 months

Despite the negative ARV effect on breast milk copper and zinc concentrations, 89–94% and 78–81%, respectively, of the milk samples obtained at 2 weeks met the concentrations used to set the AI for infants. When no ARVs were provided, 88% of the samples reached adequate zinc concentrations at 2 weeks versus 78–81% when ARV were taken, reiterating the already noted negative influence of ARV on zinc levels in breast milk at early stages of lactation. Only 38–48% of the iron concentrations at 2 weeks met AI values. However, the naturally occurring decrease in concentrations during lactation (Brown et al., 2009; Lönnerdal, Keen, & Hurley, 1981a; Silvestre et al., 2001) was lower for iron than for copper and zinc, suggesting mineral‐specific kinetic rates of transport and secretion into milk. Iron, copper, and zinc concentrations in milk are at their peak during the first few days post‐partum and decrease as lactation progresses. However, the concentrations of these trace minerals are remarkably stable at all lactation stages as they are tightly regulated and maintained by the mammary epithelial cells (Montalbetti et al., 2014).

Infants possess extensive iron stores in the liver that offset the low milk levels and ensure adequate supply. Due to the extreme changes in concentrations of minerals in milk within the first 6 months post‐partum, a range of AI values would be more accurate for estimating adequate intake than the current single value approach; the natural decrease in concentration will result in theoretically insufficient concentrations of these minerals in breast milk, even though the infant may actually be adequately supplied. Moreover, the AI values for these trace minerals are based on limited data (9–15 studies with low numbers of participants; Institute of Medicine, 2001). Thus, the concentrations of iron, copper, and zinc measured in our study cannot be interpreted as insufficient based solely on the current AI estimates. Longitudinal data from big data sets such as the BAN study provide important additional information for the assessment of milk mineral content.

### 4.3 Correlations of maternal and infant biomarkers and characteristics with human milk minerals

In the BAN study, changes in maternal TfR and Hb were associated with those observed in infant TfR and Hb (Widen et al., 2014). However, neither of these maternal or infant iron status biomarkers showed a significant correlation with milk iron, copper, or zinc, indicating that the breast milk minerals are not the determining factor for infant iron (TfR, ferritin, Hb) status during lactation. In fact, Mahdavi et al. (2010) showed that milk iron, copper, and zinc concentrations and the infant's weight for age and height for age z‐score are not significantly associated, and Murray, Murray, Murray, and Murray (1978) reported no significant differences between milk iron and infant iron status measures, showing the independence of infant outcomes from human milk mineral concentrations.

Interestingly, we found that women with lower Hb during pregnancy (≤30 weeks gestation) produced milk with higher iron concentrations at both 2 and 6 weeks, whereas none of the post‐partum maternal iron status biomarkers (Hb, ferritin, TfR) were associated with milk minerals at these TPs, suggesting that maternal iron status during pregnancy may affect milk iron concentrations during early stages of lactation. Using BeWo cell layers as a model for placental iron transfer in rats fed decreasing iron levels Gambling et al. (2001) found increased mRNA and protein expression of TfR and of the divalent metal transporter‐1 (DMT‐1). Recently, Jobarteh et al. (2017) confirmed an upregulation of mRNA of placental TfR1 in Gambian women with low iron status; this increase might represent a mechanism to ensure foetal requirements are met. Such alterations in iron metabolism could also occur in the mammary epithelial cells that regulate mineral secretion into milk, which also involves TfR‐ and DMT1‐regulated processes (Montalbetti et al., 2014). Given that this relationship was observed in two independent groups and time of pregnancy during screening did not affect Hb status (data not shown), this consistent significant and negative association between pregnancy Hb and human milk iron deserves further investigation. Parity and maternal BMI were the only characteristics associated with breast milk minerals; whereas parity and iron concentrations were correlated at 2 weeks, maternal BMI and zinc concentrations were correlated at 24 weeks. These findings indicate that higher parity could adversely affect iron, whereas higher BMI might positively influence breast milk zinc concentration during later stages of lactation.

The strongest correlations (all positive) were found among the human milk minerals. All of them were also associated with the milk fat, an observation previously described by Finley, Lönnerdal, Dewey, and Grivetti (1985). Given that all three minerals are partly present in the lipid fraction of the milk (Donangelo et al., 1989; Fransson & Lönnerdal, 1980, 1982, 1984; Hirai et al., 1990; Linder et al., 1998; Lönnerdal, Hoffman, & &Hurley L., 1982; Wooten, Shulze, Lancey, Lietzow, & Linder, 1996), the correlation with milk fat is not unexpected. Copper and zinc are both bound to serum albumin, casein, and citrate in milk (Lönnerdal et al., 1982); their positive association suggests that the two minerals are not competing for the same binding site but rather for the availability of additional binding capacity (Finley et al., 1985; Lönnerdal, Keen, & Hurley, 1981b). Positive correlations between milk iron and copper have also been reported by Feeley et al. (1983), concluding that milk from mothers with high amounts of iron may also be more likely to contain high concentrations of copper. Given the intertwined relationships of mineral metabolism (Institute of Medicine, 2001), the close association of their concentrations in milk is not surprising.

### 4.4 Associations of human milk iron, copper, and zinc with maternal and infant biomarkers, stratified by maternal Hb (anaemia status) during pregnancy and post‐partum

Limited research has focused on the relationship of maternal and infant iron status biomarkers with human milk mineral content. Kumar, Rai, Basu, Dash, and Singh (2008) showed that severe maternal anaemia adversely affected cord blood and human milk iron concentrations. In our study, moderately anaemic women with higher ferritin also had a higher concentration of iron in their milk compared to non‐anaemic or only mildly anaemic women at 2 weeks, whereas lower TfR was associated with higher milk iron content in moderately anaemic compared to non‐anaemic women at 2 and 24 weeks. Although iron secretion is tightly regulated and maintained by the mammary epithelial cells, little is known about the molecular and cellular mechanisms regulating mineral concentrations in the milk (Montalbetti et al., 2014). Thus, although milk iron concentrations are assumed to be independent from maternal intake, our observations indicate the possibility of a less understood yet complex relationship on the molecular and cellular level between maternal iron status during pregnancy and the amounts of iron in milk during early lactation.

At 6 weeks, mildly anaemic women with higher concentrations of the inflammation biomarkers AGP and CRP also had higher concentrations of iron, copper, and zinc in their milk than women with normal Hb. Based on the categories of inflammation proposed by Thurnham et al. (2008), about 23% of the mildly anaemic women at 6 weeks showed early stages of infection or convalescence. After adjusting for inflammation, the described relationships persisted, suggesting that the association of maternal AGP/CRP and milk minerals in mildly anaemic women is not directly caused by inflammation (Table S5).

The degree of maternal anaemia during pregnancy or lactation may also affect the relationship between breast milk minerals and infant biomarkers. We found that infants from mildly anaemic mothers at 2 weeks, who secrete more copper into milk, have lower AGP concentrations, an effect also found for breast milk iron and copper at 6 weeks for women who were mildly anaemic during pregnancy. Infant TfR also seemed to be affected by maternal Hb status and was lower when breast milk iron, copper, and zinc were higher, but only when comparing moderately anaemic to non‐anaemic mothers. To our knowledge, no previous reports have investigated these associations.

These relationships between milk minerals and infant status may be linked to maternal anaemia status. Alterations in maternal mineral metabolism related to maternal iron status have been recently described (Jobarteh et al., 2017); however, more research is necessary to understand any influence of this effect on infant status.

### 4.5 Human milk iron, copper, and zinc concentrations and inflammation

Information is sparse regarding the impact of inflammation on human milk minerals. However, effects of inflammation on maternal status may indirectly affect breast milk mineral concentrations. Inflammation has been associated with reduced iron incorporation into haem, and increased blood copper, and decreased zinc concentrations (Aggett & Harries, 1979; Beshgetoor & Hambidge, 1998; Van den Broek & Letsky, 2000). The leucocyte endogenous mediator is thought to be involved by enhancing the hepatic uptake of zinc and iron and by synthesis of acute phase proteins such as ceruloplasmin, a multi‐copper ferrioxidase (Gambling, Andersen, & McArdle, 2008). Recent findings describe an upregulation of placental iron and zinc transporters in Gambian women with low iron and zinc status (Jobarteh et al., 2017), proteins also located in the mammary gland (McCormick, Hennigar, Kiselyov, & Kelleher, 2014; Montalbetti et al., 2014). These alterations in mineral metabolism could extend into milk secretion, for example, due to a signalling cascade. Our findings of higher milk iron, copper, and zinc concentrations in women with inflammation indicate higher influx of these minerals into milk when inflammation occurs. Moreover, the inflammation‐related higher milk iron concentrations at 2 weeks when the woman's pregnancy Hb status was lower, and higher milk zinc concentrations at 6 weeks when their pregnancy Hb status was higher, support a potential upregulated iron and zinc secretion into milk when women were subjected to health challenges such as low status and inflammation. Interestingly, no such relationships were found with Hb status during lactation, indicating that pregnancy Hb status might have a stronger impact than Hb status during lactation. However, Van den Broek and Letsky (2000) also found an independent effect of HIV‐infection on Hb concentrations that was not associated with concurrent infection or dietary deficiencies. Thus, the extent of the interrelationships of inflammation, pregnancy Hb, and milk mineral concentrations has yet to be determined.

### 4.6 Limitations and conclusions

Despite the extensive number of milk samples analysed in the BAN study, the samples collected are opportunistic and may not reflect a 24 h milk sample. Moreover, our initial samples were collected 2 and 6 weeks into the intervention, thus not reflecting a true baseline. The low dose of copper in the LNS (only 23% of the Recommended Dietary Allowance (RDA)) may also be insufficient to detect a difference in milk copper concentrations. Exploring the data for associations based on maternal Hb status resulted in a few subgroups with small sample sizes (e.g., *n* = 13 or 16, Figures 3 and 4; Tables S3 and S4), which may not be generalizable to the larger population. However, unique characteristics of the study include longitudinal collection of breast milk in a relatively large sample of women, high adherence to recommendations to exclusively breastfeed, and the randomized control design, which enabled analysis of the effects of LNS and ARV, and their interactions, on milk micronutrients. ARV affected milk copper and zinc concentrations and was not offset by maternal supplementation during lactation. Even though this negative effect appears to be limited and only at early lactation, the extent of the impact on the infant has yet to be determined as well as possible remedies. Although we found significant correlations between maternal biomarkers and characteristics with milk minerals, those correlations were weak, and further studies are required to gain better insight into the complex relationships between maternal and infant biomarkers and breast milk mineral concentrations.

## CONFLICTS OF INTEREST

C. M. van der Horst received grant support from Abbott Laboratories and GlaxoSmithKline. All other authors declare that they have no conflicts of interest.

## CONTRIBUTIONS

DH conducted the laboratory, data, and statistical analyses, wrote the paper, and is responsible for the final content; SSF contributed to sample, data, and statistical analysis; VLF contributed to data analysis and statistics; LSA and MEB contributed to the trial design and obtained funding for the study; LHA is responsible for the laboratory analyses conducted at the WHNRC and for the final version of the manuscript. All authors reviewed manuscript revisions and contributed to the intellectual content of the manuscript.

## Supporting information


**Figure S1.** Flow chart of study participants in the BAN study: recruitment, eligibility screening, and randomization to treatment groups. ^1^ Women were randomly assigned to receive LNS and/or ARV from 0 to 28 wk. ^2^ Breast milk was analysed only in women selected as part of a matched mother‐infant micronutrient analysis (ARV‐ antiretroviral, BAN – Breastfeeding, Antiretrovirals, and Nutrition, LNS ‐ lipid‐based nutrient supplement; mat. ‐ maternal)
**Table S1.** Composition of the lipid‐based nutrient supplement formulated for lactating women aged 19‐30y
**Table S2.** Main effects and interactions of ARV and LNS of iron, copper, and zinc at 2, 6, and 24 wk in breast milk of HIV‐infected Malawian mothers assigned to one of the four treatment arms in the BAN study
**Table S3**. Associations between breast milk iron, copper, and zinc with maternal biomarkers based on haemoglobin status during pregnancy and lactation (GLM procedure; X ‐ independent variable, Y ‐ dependent variable, CRP ‐ C‐reactive protein, AGP ‐ α‐1‐acid glycoprotein, TfR ‐ soluble transferrin receptors, n ‐ number of samples. Separate models were used for each time point and FTP and anaemia status).
**Table S4**. Associations between breast milk iron, copper, and zinc with infant biomarkers based on haemoglobin status during pregnancy and lactation (GLM procedure; X ‐ independent variable, Y ‐ dependent variable, CRP ‐ C‐reactive protein, AGP ‐ α‐1‐acid glycoprotein, TfR ‐ soluble transferrin receptors, n ‐ number of samples, not significant ‐ p‐value >0.100. Separate models were used for each time point and FTP and anaemia status).
**Table S5.** Associations of inflammation adjusted breast milk iron, copper, and zinc with maternal inflammation markers AGP and CRP in mildly anaemic women at 6wk (GLM procedure; X ‐ independent variable, Y ‐ dependent variable, CRP ‐ C‐reactive protein, AGP ‐ α‐1‐acid glycoprotein, n ‐ number of samples.
