# Supplementary material for: The effects of a lipid‐based nutrient supplement and antiretroviral therapy in a randomized controlled trial on iron, copper, and zinc in milk from HIV‐infected Malawian mothers and associations with maternal and infant biomarkers
Source: Matern Child Nutr. 2017 Aug 29;14(2):e12503. doi: 10.1111/mcn.12503 (PMC5832511; doi:10.1111/mcn.12503)
Supplement: Supplementary file 1 — Figure S1. Flow chart of study participants in the BAN study: recruitment, eligibility screening, and randomization to treatment groups. 1 Women were randomly assigned to receive LNS and/or ARV from 0 to 28 wk. 2 Breast milk was analysed only in women selected as part of a matched mother‐infant micronutrient analysis (ARV‐ antiretroviral, BAN – Breastfeeding, Antiretrovirals, and Nutrition, LNS ‐ lipid‐based nutrient supplement; mat. ‐ maternal) Table S1. Composition of the lipid‐based nutrient supplement formulated for lactating women aged 19‐30y Table S2. Main effects and interactions of ARV and LNS of iron, copper, and zinc at 2, 6, and 24 wk in breast milk of HIV‐infected Malawian mothers assigned to one of the four treatment arms in the BAN study Table S3. Associations between breast milk iron, copper, and zinc with maternal biomarkers based on haemoglobin status during pregnancy and lactation (GLM procedure; X ‐ independent variable, Y ‐ dependent variable, CRP ‐ C‐reactive protein, AGP ‐ α‐1‐acid glycoprotein, TfR ‐ soluble transferrin receptors, n ‐ number of samples. Separate models were used for each time point and FTP and anaemia status). Table S4. Associations between breast milk iron, copper, and zinc with infant biomarkers based on haemoglobin status during pregnancy and lactation (GLM procedure; X ‐ independent variable, Y ‐ dependent variable, CRP ‐ C‐reactive protein, AGP ‐ α‐1‐acid glycoprotein, TfR ‐ soluble transferrin receptors, n ‐ number of samples, not significant ‐ p‐value >0.100. Separate models were used for each time point and FTP and anaemia status). Table S5. Associations of inflammation adjusted breast milk iron, copper, and zinc with maternal inflammation markers AGP and CRP in mildly anaemic women at 6wk (GLM procedure; X ‐ independent variable, Y ‐ dependent variable, CRP ‐ C‐reactive protein, AGP ‐ α‐1‐acid glycoprotein, n ‐ number of samples. [file MCN-14-e12503-s001.docx]

**SUPPLEMENTAL MATERIAL**

**LEGENDS TO FIGURES**

**Supplemental Figure 1:** Flow chart of study participants in the BAN study: recruitment, eligibility screening, and randomization to treatment groups. ^1^ Women were randomly assigned to receive LNS and/or ARV from 0-28 wk. ^2^ Breast milk was analyzed only in women selected as part of a matched mother-infant micronutrient analysis (ARV- antiretroviral, BAN – Breastfeeding, Antiretrovirals, and Nutrition, LNS - lipid-based nutrient supplement; mat. - maternal)

**Supplemental Figure 1**


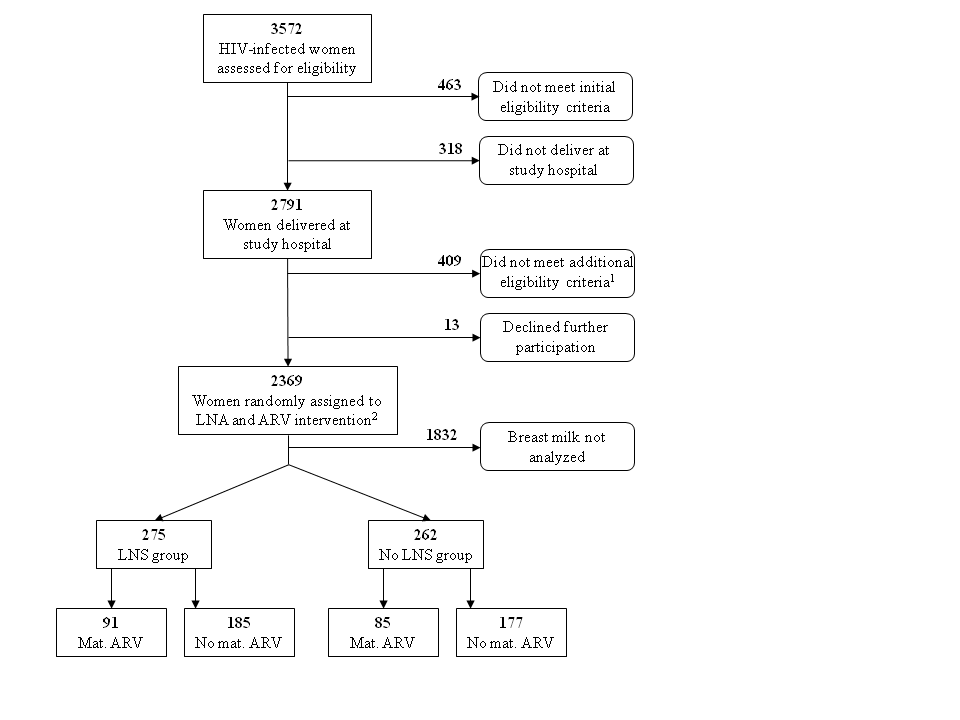


**Supplemental Table 1:** Composition of the lipid-based nutrient supplement formulated for lactating women aged 19-30y

| **Nutrient^a^** | **RDA for lactating women** | **Amount per 2 packets (140g)** |
| --- | --- | --- |
| Energy (kcal (kj)) | ---- | 746 (3120) |
| Protein (g) | ---- | 20.8 |
| Iron (mg) | 9 | 15 |
| Copper (mg) | 1.3 | 0.3 |
| Zinc (mg) | 12 | 19 |
| Calcium (mg) | 1000 | 294 |
| Magnesium (mg) | 310 | 124 |
| Potassium (g) | 5.1 | 1.1 |
| Selenium (µg) | 70 | 75 |
| Iodine (µg) | 290 | 200 |
| Phosphorus (µg) | 700 | 1200 |
| Thiamin (mg) | 1.4 | 1.6 |
| Riboflavin (mg) | 1.6 | 1.8 |
| Niacin (mg equivalent) | 17 | 20 |
| Pyridoxine (mg) | 2.0 | 2.2 |
| Folic acid (µg) | 500 | 300 |
| Vitamin B12 (µg) | 2.8 | 2.6 |
| Ascorbic acid (mg) | 120 | 100 |
| α-tocopherol (mg) | 19 | 12 |

^a^ The supplement consisted of ground peanuts, dried skim milk, vegetable fat, sugar, multivitamin-mineral premix (Nutriset, France; [www.nutriset.fr](http://www.nutriset.fr)). RDA, Recommended Dietary Allowance (Institute of Medicine).

**Supplemental Table 2:** Main effects and interactions of ARV and LNS of iron, copper, and zinc at 2, 6, and 24 wk in breast milk of HIV-infected Malawian mothers assigned to one of the four treatment arms in the BAN study

| **Treatment** |  | **Iron** | **Copper** | **Zinc** |
| --- | --- | --- | --- | --- |
| **Main effects and interaction^1^** | | **p-values** | **p-values** | **p-values** |
| LNS | | 0.66 | 0.07 | 0.28 |
| ARV | | 0.25 | 0.30 | 0.68 |
| TP | | <0.001 | <0.001 | <0.001 |
| FTP | | 0.001 | <0.001 | <0.001 |
| ARV*LNS | | 0.70 | 0.24 | 0.29 |
| ARV*TP | | 0.72 | 0.25 | 0.07 |
| ARV*FTP | | 0.31 | 0.28 | 0.031 |
| LNS*TP | | 0.13 | 0.66 | 0.40 |
| LNS*FTP | | 0.18 | 0.81 | 0.46 |
| ARV*LNS*TP | | 0.20 | 0.30 | 0.87 |
| ARV*LNS*FTP | | 0.44 | 0.95 | 0.48 |
| ARV*TP*FTP | | 0.17 | 0.034 | 0.51 |
| LNS*TP*FTP | | 0.89 | 0.71 | 0.46 |
| ARV*LNS*TP*FTP | | 0.51 | 0.49 | 0.34 |

^1^Main effects and interactions of LNS and ARV were tested by analysis of variance. Mixed model repeated measures analysis was used to test for the main effects of time as well as interactions between treatment variables and time. ARV, antiretrovirals; BAN, Breastfeeding, Antiretrovirals, and Nutrition; FTP, first time point for collection (2 or 6wk); LNS, lipid-based nutrient supplement; TP, time point (2/6 wk or 24wk).

**Supplemental Table 3**: Associations between breast milk iron, copper, and zinc with maternal biomarkers based on hemoglobin status during pregnancy and lactation (GLM procedure; X - independent variable, Y - dependent variable, CRP - C-reactive protein, AGP - α-1-acid glycoprotein, TfR - soluble transferrin receptors, n - number of samples. Separate models were used for each time point and FTP and anemia status).

| **Class** | **X** | **Y** | **Moderately anemic** | | | **Mildly anemic** | | **Non-anemic** |
| --- | --- | --- | --- | --- | --- | --- | --- | --- |
| **Maternal Biomarkers** | | | **Coefficient^a^**  **(Std Error)** | **P-value^b^** | **P-value^c^** | **Coefficient (Std Error)** | **P-value^b^** | **Coefficient**  **(Std Error)** |
| **2 weeks** |  |  |  |  |  |  |  |  |
| Anemia^e^ | Ferritin | Iron | 0.079  (0.027) | 0.037 | 0.017 | -0.005  (0.023) | 0.38 | 0.018  (0.012) |
|  | TfR | Iron | -0.136  (0.068) | 0.024 | 0.13 | -0.001  (0.058) | 0.62 | 0.031  (0.027) |
|  | CRP | Copper | -0.034  (0.010) | 0.016 | 0.12 | -0.015  (0.008) | 0.54 | -0.009  (0.004) |
| n |  |  | 41 |  |  | 49 |  | 276 |
| Anemia_Preg_^f^ | TfR | Iron | -0.065  (0.039) | 0.030 | 0.25 | 0.001  (0.043) | 0.38 | 0.050  (0.035) |
| n |  |  | 91 |  |  | 106 |  | 169 |
| 6 weeks |  |  |  |  |  |  |  |  |
| Anemia | CRP | Iron | 0.009  (0.027) | 0.91 | 0.06 | 0.083  (0.028) | 0.013 | 0.006  (0.014) |
|  | CRP | Copper | -0.001  (0.012) | 0.77 | 0.14 | 0.026  (0.013) | 0.036 | -0.005  (0.066) |
|  | AGP | Iron | 0.139  (0.134) | 0.70 | 0.07 | 0.459  (0.113) | 0.002 | 0.086  (0.037) |
|  | AGP | Copper | -0.053  (0.063) | 0.27 | 0.005 | 0.184  (0.053) | 0.004 | 0.019  (0.017) |
|  | AGP | Zinc | 0.269  (0.203) | 0.39 | 0.15 | 0.650  (0.172) | 0.002 | 0.086  (0.056) |
| n |  |  | 13 |  |  | 25 |  | 131 |
| Anemia_Preg_ | AGP | Iron | 0.305  (0.107) | 0.027 | 0.29 | 0.163  (0.079) | 0.21 | 0.051  (0.040) |
| n |  |  | 35 |  |  | 53 |  | 81 |
| 24 weeks |  |  |  |  |  |  |  |  |
| Anemia | TfR | Iron | -0.233  (0.083) | 0.008 | 0.24 | -0.124  (0.041) | 0.009 | -0.006  (0.019) |
|  | Hb_preg_ | Iron | 0.054  (0.038) | 0.12 | 0.004 | -0.085  (0.030) | 0.009 | -0.006  (0.006) |
|  | Hb_preg_ | Zinc | 0.104  (0.064) | 0.066 | 0.036 | -0.068  (0.051) | 0.32 | -0.016  (0.011) |
| n |  |  | 16 |  |  | 45 |  | 476 |
| Anemia_Preg_ |  |  |  |  |  |  |  |  |
|  | TfR | Iron | -0.081  (0.030) | 0.033 | 0.24 | -0.030  (0.031) | 0.39 | 0.006  (0.028) |
| n |  |  | 126 |  |  | 160 |  | 251 |

^a^ Coefficient obtained from regression procedure. ^b^P-values for comparison of moderately or mildly anemic to non-anemic group using linear regression analysis. ^c^P-values for comparison of moderately to mildly anemic groups using linear regression analysis. ^d^Anemia, state of anemia - moderately, mild, or non-anemic - based on maternal hemoglobin status at indicated week postpartum (World Health Organization 2003). ^e^Anemia_Preg_, state of anemia during pregnancy (moderately, mild, or non-anemic based on maternal Hb during pregnancy, World Health Organization, 2003).

**Supplemental Table 4**: Associations between breast milk iron, copper, and zinc with infant biomarkers based on hemoglobin status during pregnancy and lactation (GLM procedure; X - independent variable, Y - dependent variable, CRP - C-reactive protein, AGP - α-1-acid glycoprotein, TfR - soluble transferrin receptors, n - number of samples, not significant - p-value > 0.100. Separate models were used for each time point and FTP and anemia status).

| **Class** | **X** | **Y** | **Moderately anemic** | | | **Mildly anemic** | | **Non-anemic** |
| --- | --- | --- | --- | --- | --- | --- | --- | --- |
| **Infant Biomarkers** | |  | **Coefficient^a^**  **(Std Error)** | **P-value^b^** | **P-value^c^** | **Coefficient (Std Error)** | **P-value^b^** | **Coefficient**  **(Std Error)** |
| **2 weeks** |  |  |  |  |  |  |  |  |
| Anemia^d^ | Copper | AGP | -0.558  (0.849) | 0.47 | 0.23 | -2.13  (0.988) | 0.037 | 0.125  (0.434) |
| N |  |  | 41 |  |  | 49 |  | 276 |
| Anemia_Preg_^e^ | Zinc | TfR | 0.207  (0.177) | 0.037 | 0.60 | 0.079 (0.167) | 0.11 | -0.282  (0.153) |
| n |  |  | 91 |  |  | 106 |  | 169 |
| **6 weeks** |  |  |  |  |  |  |  |  |
| Anemia | Iron | Ferritin | 0.788  (2.49) | 0.95 | 0.42 | -1.35  (0.786) | 0.018 | 0.945  (0.545) |
| n |  |  | 13 |  |  | 25 |  | 131 |
| Anemia_Preg_ | Iron | TfR | -0.801  (0.315) | 0.004 | 0.32 | -0.329  (0.359) | 0.08 | 0.527  (0.325) |
|  | Copper | TfR | -1.30  (0.772) | 0.016 | 0.23 | 0.028  (0.788) | 0.28 | 1.12  (0.626) |
|  | Zinc | TfR | -0.622  (0.256) | 0.018 | 0.39 | -0.320  (0.237) | 0.13 | 0.138  (0.188) |
|  | Iron | AGP | -0.158  (0.289) | 0.24 | 0.34 | -0.571  (0.323) | 0.041 | 0.335  (0.299) |
|  | Copper | AGP | -0.292  (0.698) | 0.60 | 0.17 | -1.65  (0.698) | 0.043 | 0.186  (0.566) |
| n |  |  | 35 |  |  | 53 |  | 81 |
| **24 weeks** |  |  |  |  |  |  |  |  |
| Anemia | Copper | CRP | 11.5  (4.83) | 0.009 | 0.046 | 0.885  (2.20) | 0.34 | -1.35  (0.769) |
| n |  |  | 16 |  |  | 45 |  | 476 |

^a^ Coefficient obtained from regression procedure. ^b^P-values for comparison of moderately or mildly anemic to non-anemic group using linear regression analysis. ^c^P-values for comparison of moderately to mildly anemic groups using linear regression analysis. ^d^Anemia, state of anemia - moderately, mild, or non-anemic - based on maternal hemoglobin status at indicated week postpartum (World Health Organization 2003). ^e^Anemia_Preg_, state of anemia during pregnancy (moderately, mild, or non-anemic based on maternal Hb during pregnancy, World Health Organization, 2003).

**Supplemental Table 5:** Associations of inflammation adjusted breast milk iron, copper, and zinc with maternal inflammation markers AGP and CRP in mildly anemic women at 6wk (GLM procedure; X - independent variable, Y - dependent variable, CRP - C-reactive protein, AGP - α-1-acid glycoprotein, n - number of samples.

| **Class** | **X** | **Y** | **Moderately anemic** | | | **Mildly anemic** | | **Non-anemic** |
| --- | --- | --- | --- | --- | --- | --- | --- | --- |
| **Maternal Biomarkers** | | | **Coefficient^a^**  **(Std Error)** | **P-value^b^** | **P-value^c^** | **Coefficient (Std Error)** | **P-value^b^** | **Coefficient**  **(Std Error)** |
| **6 weeks** |  |  |  |  |  |  |  |  |
| Anemia^d^ | CRP | Iron | -0.009  (0.027) | 0.91 | 0.06 | -0.083  (0.028) | 0.013 | -0.006  (0.014) |
|  | CRP | Copper | 0.001  (0.012) | 0.77 | 0.14 | -0.026  (0.013) | 0.034 | 0.004  (0.006) |
|  | AGP | Iron | 0.139  (0.134) | 0.70 | 0.07 | 0.459  (0.113) | 0.002 | 0.086  (0.037) |
|  | AGP | Copper | -0.053  (0.063) | 0.27 | 0.005 | 0.184  (0.053) | 0.004 | 0.019  (0.017) |
|  | AGP | Zinc | 0.269  (0.203) | 0.39 | 0.15 | 0.650  (0.172) | 0.002 | 0.086  (0.056) |
| n |  |  | 13 |  |  | 25 |  | 131 |

^a^ Coefficient obtained from regression procedure. ^b^P-values for comparison of moderately or mildly anemic to non-anemic group using linear regression analysis. ^c^P-values for comparison of moderately to mildly anemic groups using linear regression analysis. ^d^Anemia, state of anemia - moderately, mild, or non-anemic - based on maternal hemoglobin status at indicated week postpartum (World Health Organization 2003).
